# Exploring the Effects of Lifestyle Disruptions on Physical Fitness in Children and Adolescents: a Systematic Scoping Review

**DOI:** 10.1186/s40798-025-00883-0

**Published:** 2025-06-07

**Authors:** Lou Dambel, Giovanna Del Sordo, Oussama Saidi, Pascale Duché

**Affiliations:** 1 Laboratoire Jeunesse-Activité Physique et Sportive-Santé (J-AP2S), Toulon University, Toulon, F-83041 France; 2 Psychology Department, New Mexico State University, 1780 E University Blvd, Las Cruces, NM 88003 USA

**Keywords:** Physical Fitness, Lifestyle Disruption, Children, Adolescents, Health

## Abstract

**Background:**

The objective of this scoping review was to systematically summarize the available literature investigating the impact of various lifestyle disruptions—including lockdowns, school vacations, and training cessation—on the physical fitness components of children and adolescents aged 4–18 years.

**Methods:**

A search for relevant studies was conducted across PubMed and ScienceDirect databases (until May 2024). Study selection and data extraction were independently performed by two reviewers using the Cadima website. A graphical analysis was conducted to present the findings of the included studies based on the effects of each lifestyle disruption on physical fitness components, such as cardiorespiratory fitness, muscle strength, explosive strength or power, speed, agility, balance, and flexibility.

**Results:**

A total of 223 records were initially identified, with 60 studies meeting the inclusion criteria for analysis. The studies assessed the impact of lockdowns (*n* = 8), school vacations (*n* = 16), and training cessation (*n* = 36) on various physical fitness components. The results indicated consistent declines in cardiorespiratory fitness, particularly among older adolescents, during these disruptions. In contrast, muscle strength and power remained relatively stable.

**Conclusion:**

Lifestyle disruptions have a notable effect on physical fitness in children and adolescents. While different types of disruptions exert varying effects, all appear to significantly affect young populations. Further research is needed, particularly focusing on girls and incorporating better control of health-related behaviors during these periods. Understanding the long-term consequences and developing strategies to support and maintain youth fitness during such disruptions should be a priority.

**Supplementary Information:**

The online version contains supplementary material available at 10.1186/s40798-025-00883-0.

## Key Points

• Lifestyle disruptions (COVID-19 lockdown, school vacations, and training cessation) have a noticeable impact on physical fitness in children and adolescents, with effects varying by the type and duration of the disruption.

• The review found consistent declines in cardiorespiratory fitness, while muscle strength showed more stability. However, the impact varied across different age groups.

• More research is needed to explore the impact of gender and improve monitoring of physical activity, sedentary behavior, and sleep during lifestyle disruptions.

## Background

In response to the global health crisis triggered by the coronavirus disease outbreak, public policies faced an unprecedented challenge in implementing effective measures. These measures included movement restrictions, social distancing, and the closure of communal spaces such as schools, supermarkets, and sports clubs. However, given the lack of existing data, the decision to implement more severe measures (i.e., lockdowns) raised critical questions about their short- and long-term effect on health, especially among young people.

The abrupt cessation of school, sport and routine activities revealed the vulnerability of children and adolescents to sudden changes in their structured environment [1, 2]. Several studies have reported effects of COVID-19 induced disruptions on body mass [3], anxiety and mental health [4]. In addition, numerous studies have sought to assess the effect of these disruptions on children’s health behaviors, revealing significant changes in physical activity, sedentary behaviors, sleep, and diet [2, 5]. A scoping review conducted in 2021, including 150 studies, consistently reported declines in physical activity time, increases in screen time and total sedentary behavior, shifts to later bed and wake times, and increases in sleep duration in children and adolescents [6].

The pandemic induced significant disruptions in the daily lives of individuals, families, and communities [4]. Godber and Atkins [7] classify these disruptions into four main areas: (i) social lifestyle, encompassing changes or interruptions in evolving spaces and social interactions; (ii) learning, characterized by disruptions in educational processes, such as school closures; (iii) active lives, involving disturbances in patterns of physical activity, sedentary behaviors, and exercise; and (iv) livelihood, referring to interruptions or alterations in individuals’ means of earning income. According to this definition, other situations beyond the COVID-19 lockdown—such as school vacations, training cessation, imprisonment, or bed rest due to injury or illness—also qualify as disruptions. It is therefore essential to examine the effects of these various disruptive situations on the lifestyle and health behaviors (physical activity, sedentary behaviors, and sleep) of children and youth.

Lifestyle behaviors are associated with physical fitness [8, 9]. Physical fitness, defined as the physical ability to engage in physical activity, comprises a spectrum of components such as cardiorespiratory fitness, muscular strength and power, endurance, speed, flexibility, agility, and balance [10]. Surprisingly, despite being a key health predictor, the effects of lockdown on physical fitness have received limited attention [11–14], with most of the studies addressing it being cross-sectional. Among these few, declines were reported in aerobic fitness and muscle strength during these restrictive periods [15–17].

Students experience weeks of vacations annually, facing substantial disruptions during school closures. The “structured days hypothesis” [18] proposes a research framework suggesting that unstructured vacation days may lead to increased sedentary behavior and reduced physical activity levels. Similar to lockdown, recent research reports an increase in body mass [19, 20] over vacations, but studies exploring other components of physical fitness remain very scarce.

Training cessation, as defined by Bosquet et Mujika [21], refers to a temporary discontinuation or complete abandonment of a systematic program of physical conditioning, therefore also inducing disruptions [7]. In adults, the physiological adaptations induced by training and their loss through training cessation are both dependent on the duration of the cessation period and the baseline levels of the participants [22, 23]. In the literature on young populations, findings regarding the effects of training cessation are inconsistent. Studies indicate the maintenance of physical fitness [24] while others report a significant decrease in the assessed components [25].

Due to the absence or very small number of studies investigating physical fitness before and after a period of incarceration or bed rest in young population, the present analysis focuses on three primary lifestyle disruptions: lockdown, vacations, and training cessation. The aims of this scoping review are twofold: (1) to provide an overview of studies assessing physical fitness changes in young individuals during different lifestyle disruptions, (2) to identify potential moderators such as duration of the disruption and age and gender of the participants.

## Methods

This systematic scoping review followed the PRISMA (Preferred Reporting Items for Systematic Reviews and Meta-Analyses) guidelines for reporting in systematic reviews [26]. This review was not registered.

### Inclusion Criteria

Studies that investigated the effects of lifestyle disruptions as defined by Godber et Akins [7] on physical fitness in children and adolescents were analyzed. The inclusion of studies followed the pre-determined population, intervention/exposure, comparator, outcome, and type of study (PICOT) criteria [27].

#### Population

Children and adolescents, girls, and boys (aged 4–18 years), without restrictions on clinical diagnosis, or on physical fitness levels. Infants and toddlers (aged 0–3 years) were excluded from the review due to their distinct movement behaviors and physical fitness characteristics [28, 29]. Studies were eligible for inclusion in the full-text review if they encompassed populations both within and outside the 4–18-year age range, if results were segmented by age. Additionally, studies with overlapping age ranges were considered, if most of the sample fell within the specified age range. In this review, we focused on the 4–18 years age group as our population of interest. However, some of the included studies also evaluated a broader age range [30–32] but were still included due to their relevance to our research questions.

#### Intervention/exposure

Any articles that investigated physical fitness within the context of disruptive situations affecting young populations. “Lifestyle disruptions” represent situations that induced one of the disruptions identified by Godber et Atkins [7]. “Lockdown” is the temporary condition imposed by governmental authorities during the outbreak of COVID-19 in which people are required to stay in their homes and refrain from or limit activities [30]. This definition distinguishes a lockdown from the broader concept of the ‘COVID-19 pandemic’. “Vacations” refer to periods when schools and colleges are closed, such as summer vacations. This definition excludes individuals who participate in camps or school activities during vacation periods. “Training cessation” is a temporary discontinuation or complete abandonment of a systematic program of physical conditioning [21].

#### Comparator

Pre- and post-measures (before and after lockdown, vacations, and/or training cessation) in the same sample at different timepoints.

#### Outcomes

Any assessment of physical fitness components, including cardiorespiratory fitness, muscle strength and endurance, explosive power, speed, power-strength, flexibility, agility, and balance. These components had to be assessed using validated scales, questionnaires, or tests. Anthropometric measures were excluded from this review, as their effects had already been extensively investigated in previous studies addressing lockdowns, school vacations, and training cessation [3, 18, 31].

#### Type of Study

Only studies available in English and French were considered for inclusion. All pre-post longitudinal designs were considered for inclusion. Cross-sectional studies, reviews, meta-analyses, conference abstracts and protocols were excluded.

### Search Strategy and Study Selection

**Fig. 1:**
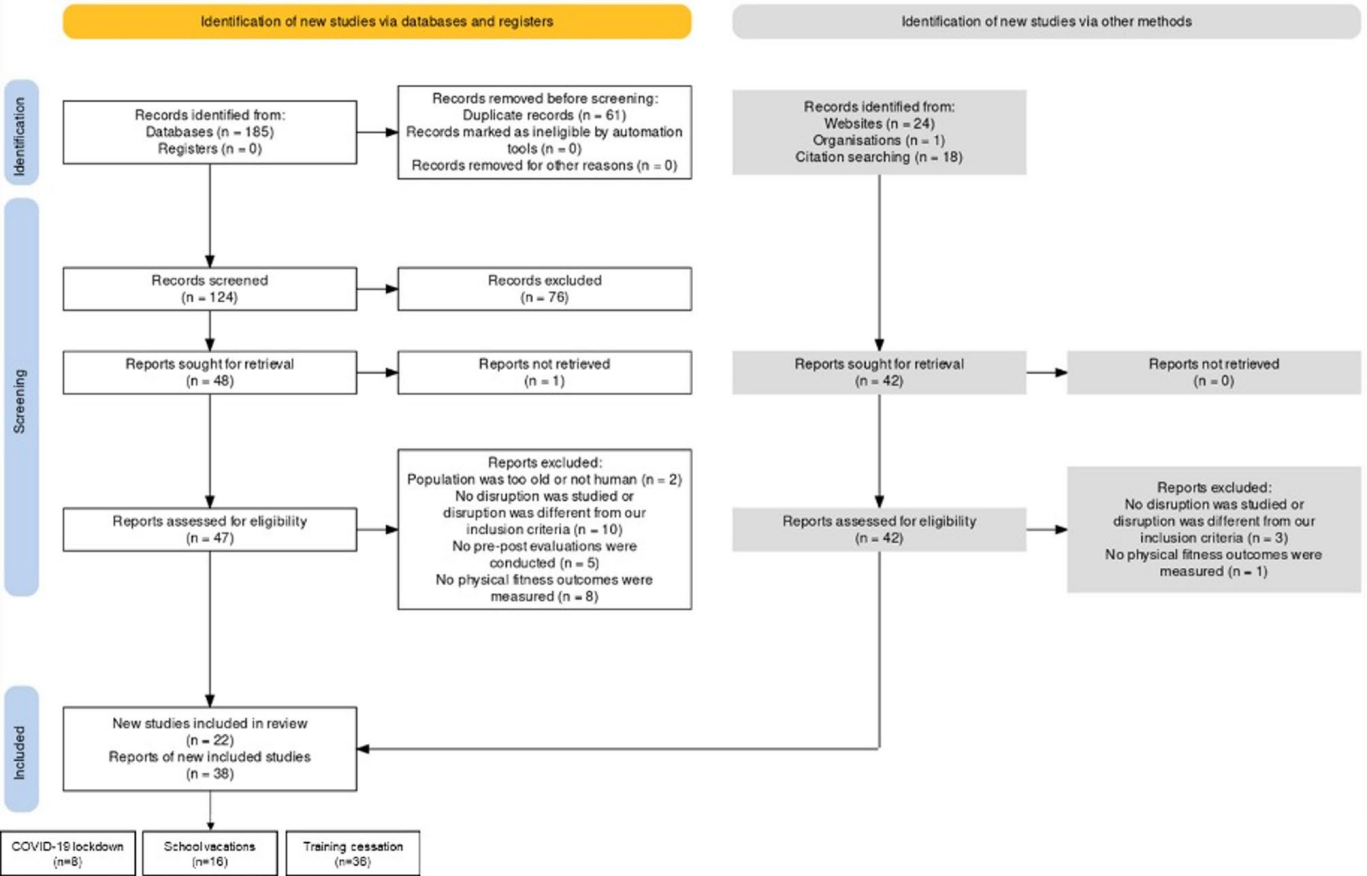
PRISMA Flow chart of study literature search. (Page MJ, McKenzie JE, Bossuyt PM, Boutron I, Hoffmann TC, Mulrow CD, et al. The PRISMA 2020 statement: an updated guideline for reporting systematic reviews. BMJ 2021;372:n71. doi: 10.1136/bmj.n71)

The searches were conducted until May 2024. Papers published after June 1, 2024, were excluded from the final review. The searches were performed in the PubMed and ScienceDirect databases, and the reference lists of all selected articles were manually examined to identify any additional relevant studies. Due to the limited indexing of relevant articles, we complemented our search by exploring Google Scholar and general web sources to ensure comprehensive coverage of the literature and minimize potential biases (Fig. 1).

Study selection was facilitated using the Cadima website [32] as an evidence synthesis tool, with references lists imported for screening. Two reviewers (LD and GD) independently screened the titles and abstracts of the studies for eligibility and later retrieved full-text article. Two reviewers (LD and GD) then determined the eligibility of each study in accordance with inclusion and exclusion criteria. Any disagreements were discussed as a group (LD, GD, and PD) until consensus was reached. The full search strategy can be found in Appendix S1.

### Data Extraction

Data extraction was carried out following the completion of screening. Two reviewers (LD and GD) extracted the included articles from the Cadima website and compiled them in an Excel file. Subsequently, another reviewer (PD) independently verified the accuracy of the extracted data. The following information was extracted from the included studies: (1) study details including authors, year of publication and country; (2) study design and aim; (3) population characteristics such as age, gender, and stage of maturation; (4) study procedure; (5) nature and duration of the disruption; types and characteristics of interventions/exposures; (6) outcome measures (physical fitness tests); and (6) results. The complete data extraction form can be found in Appendix S2.

### Assessment of Studies’ Quality

Quality and risk of bias were assessed using three National Institute of Health tools for observational, before-after, and controlled intervention studies (NIH, 2021a, 2021b, 2021c). Each study received a global numerical quality score and an overall judgment (poor, fair, good). Scores below 55% were “poor,” 55–75% were “fair,” and above 75% were “good.” Two reviewers (LD and GD) independently assessed each study, resolving any disagreements through group discussion (LD, GD, PD). Full details are available in Appendices S5, S6 and S7.

### Analysis Strategy

A meta-analysis of the selected studies was not conducted due to the high heterogeneity of outcome measures, interventions, study designs, and populations. Instead, a narrative analysis was conducted following the SWiM (Synthesis Without Meta-analysis) guidelines to report results [33]. Studies were categorized based on the type of lifestyle disruption and the physical fitness components affected. Given the varied terminologies in the literature, we recorded the specific tests used and the components they assessed, retaining the original authors’ classifications for unique or unfamiliar tests (Appendices S2, S3 and S4).

To enhance precision, outcomes were treated as individual data points, allowing multiple data points from a single study. When available, pre-, and post-values were converted to percentage changes; otherwise, only the effect direction (increase, decrease, or no change) was recorded. Then, the effects of disruptions were visualized by a traffic light graphical representation: red for “decrease,” yellow for “no change,” and green for “increase” in physical fitness. “Increase” indicates significant improvement, while “decrease” reflects a significant decline, such as a longer sprint time. “No change” indicates the lack of a statistically significant increase or decrease from pre- to post-disruption data, as reported by the authors. The effect of moderators (age, gender, duration) on physical fitness changes were graphically analyzed. Data points were included if they provided numerical values before and after disruptions, with sufficient data (> 50 points) required for inclusion, except for gender, where data on girls were insufficient. For these analyses, pre-post changes were expressed as percentages. Additionally, the baseline effect on physical fitness evolution was analyzed using data points from identical parameters measured by the same test (> 20 data points per parameter).

## Results

### Study Selection

The electronic search identified 223 potentially eligible reports: 185 from PubMed (*n* = 94) and ScienceDirect (*n* = 91). After removing duplicates, 124 records were screened at the title/abstract level, with 76 studies excluded. An additional 43 eligible records were found through website and reference list searches. Of the 89 full-text articles retrieved, 60 publications were considered relevant to evaluating the effect of lifestyle disruption on physical fitness. The study selection process is shown in Fig. 1.

### Characteristics of the Included Studies

Eight studies analyzed the impact of lockdown on physical fitness [34–41], while 16 studies explored the effects of school vacations [42–57] and 36 studies investigated the effects of training cessation [24, 25, 58–91], The duration of these disruptive situations ranged from 2 to 40 weeks. All studies that focused on the effect of school vacations specifically analyzed summer vacations. Sample sizes varied from 7 to 27,181 participants, with ages ranging from 7 to 19 years. The gender distribution was 38% boys (*n* = 23), 7% girls (*n* = 4), and 55% mixed (*n* = 32) (Appendices S2, S3, and S4).

**Fig. 2:**
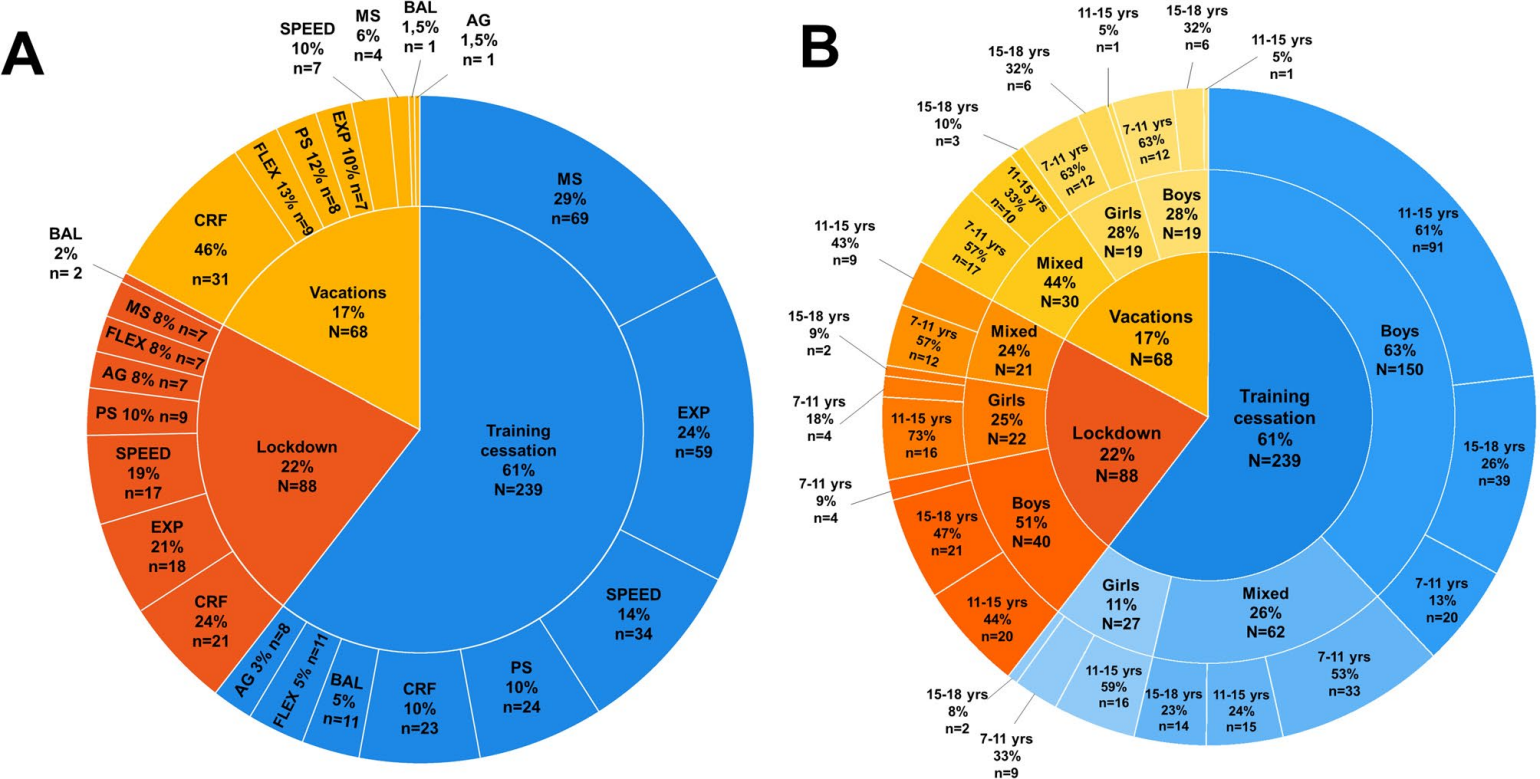
Breakdown of data points. (**Panel A**) Breakdown by disruptive situation and by physical fitness component. (**Panel B**) Breakdown by disruptive situation, gender, and age range

Each study measured one or more physical fitness (PF) components, resulting in a total of 395 data points. Each data point represented an assessment of a unique PF component for one population group, pre- and post-disruption. For lockdowns (*n* = 88; 22%), PF components were measured in balanced proportions, with cardiorespiratory fitness (CRF) being the most frequently assessed component (*n* = 21; 24%). During vacations (*n* = 68; 17%), CRF was also the most studied component (*n* = 31; 46%). In studies investigating training cessation (*n* = 239; 61%), muscle strength (MS) was the most frequently analyzed component (*n* = 69; 29%) (Fig. 2, Panel A).

Boys were the primary focus in both lockdown (*n* = 40; 51%) and training cessation (*n* = 150; 63%) studies. In school vacation studies, gender distribution was more balanced, with mixed-gender groups being the most frequently assessed (*n* = 30; 44%) (Fig. 2, Panel B).

Across the studies, 108 different tests were used to assess eight physical fitness components, with 32 methods specifically for muscle strength (Appendix S8).

### Methodological Quality Assessments

The quality assessment results, summarized in Appendices S5, S6 and S7, utilized three different quality scales based on study design. Among 32 pre-post studies without control groups [25, 35, 40, 42–45, 48, 50–54, 57, 60, 61, 63, 69, 70, 73–75, 78, 80, 81, 84, 87, 88, 90–93], 87.5% were rated as good quality, with the main issue being unclear reporting [e.g., eligibility criteria, participant enrollment). Of the 22 controlled intervention studies [24, 47, 49, 56, 58, 59, 62, 64–68, 71, 72, 76, 77, 79, 82, 83, 85, 86, 89], only 4 were rated as good quality, with 54.5% rated as fair and 6 as poor, primarily due to inadequate reporting (e.g., randomization, treatment allocation, blinding, power analysis, and statistical analysis). Six cross-sectional or cohort studies were assessed [36–39, 41, 46], with 83% rated as good quality and one as poor, mainly due to missing power analyses, lack of blinding, and inadequate control of confounding variables.

### Effect of Lockdown on Physical Fitness Components

**Fig. 3:**
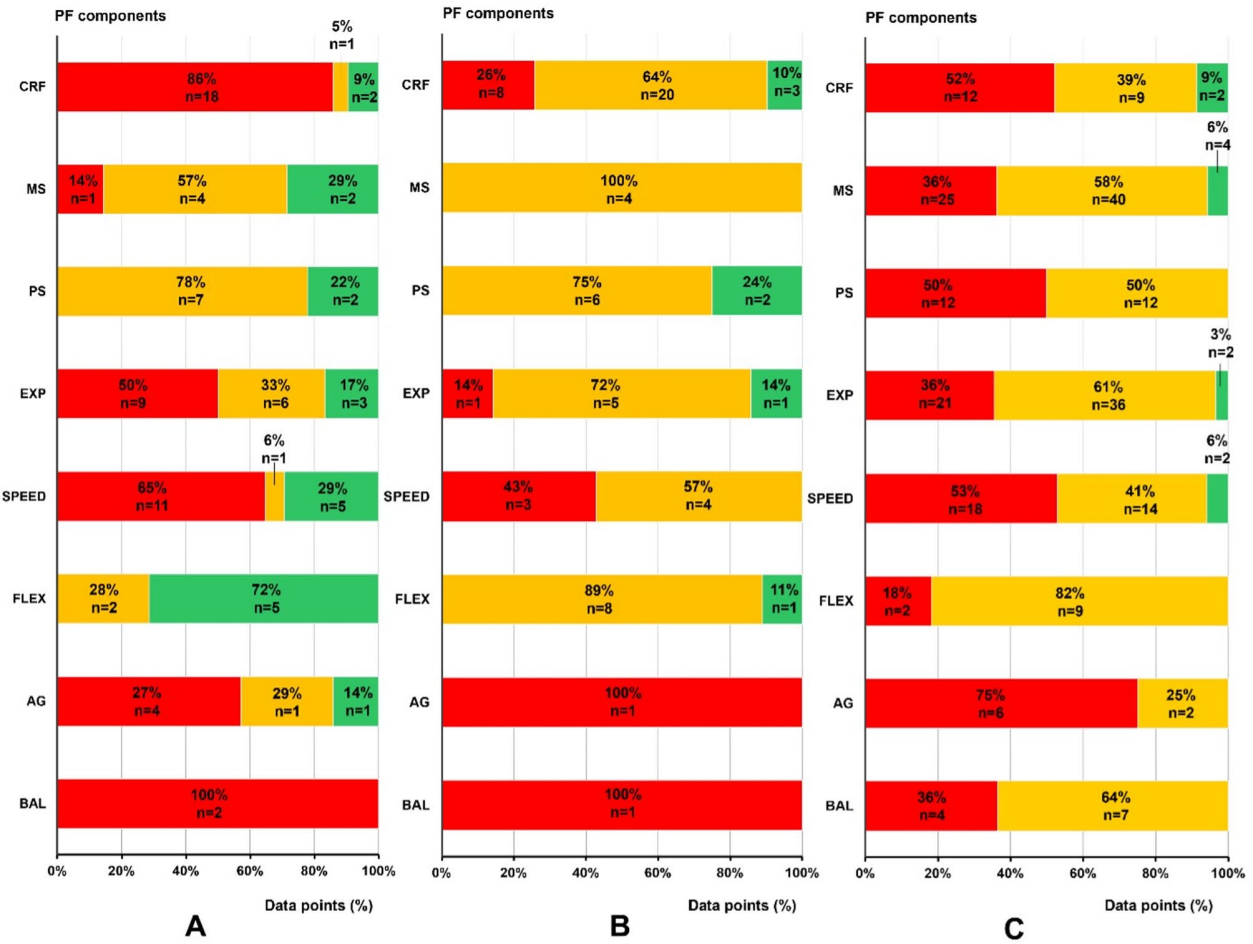
Traffic light analysis of pre- to post-disruption changes on physical fitness (PF) components. **(Panel A)** From pre- to post-lockdown. **(Panel B)** From pre- to post-vacations. **(Panel C)** From pre- to post- training cessation *The bars represent the percentage of data points reporting significant increases (green), significant decreases (red), or no changes (yellow) in physical fitness components from pre- to post-disruption periods **CRF: cardiorespiratory fitness, MS: muscle strength, EXP: explosive power, PS: power-strength, FLEX: flexibility, AG: agility, BAL: balance

Eight studies [34–41] evaluating effects of lockdown on physical fitness in children provided 88 data points, with participants aged 6 to 22 years and sample sizes ranging from 31 to 27,681. Boys were the most assessed (*n* = 45; 51%), followed by girls (*n* = 22; 25%) and mixed-gender groups (*n* = 21; 24%). The duration of lockdown varied from 6 to 40 weeks (Fig. 3, Panel A).

#### Cardiorespiratory Fitness

Six studies [34, 36–38, 40, 41] yielded 21 data points, making CRF the most frequently assessed component. Ten data points were obtained in males, 6 in females, and 5 in mixed gender groups. Most data points (*n* = 18; 86%) reported decreases in CRF, with reductions of up to 43%, primarily observed during lockdowns lasting 12 to 36 weeks. Two data points showed increases, and one reported no significant change. Among the 16 tests employed to assess CRF, he 20-m shuttle run test was the most used method (*n* = 6) (Appendix S8).

#### Muscle Strength

Seven data points from 2 studies [36, 40] studied muscle strength, with 3 data points in mixed gender groups and 2 in both males and females. The handgrip test was used in 6 out of 7 data points. No significant changes were observed in 4 data points (57%), while 2 data points (29%) reported improvements of up to 6%. One data point indicated a decrease after 24 weeks of lockdown.

#### Power-strength

Three studies [37, 38, 41] contributed 9 data points assessing power-strength. Four data points studied males and females separately, and one data point a mixed-gender group. Assessment methods included pull-ups to failure (*n* = 1), sit-ups within a minute (*n* = 5), and chin-ups within a minute (*n* = 3) (Appendix S8). No decreases were observed; 2 data points showed increases, and 7 data points (78%) reported no significant changes.

#### Explosive Strength or Power

Seven studies [34–36, 38–41] contributed 18 data points on explosive strength or power (EXP), with 61% of data points (*n* = 11) in males, 22% (*n* = 4) in females, and 17% (*n* = 3) in mixed-gender groups. Four different tests were used: the standing broad jump (*n* = 11), and the countermovement jump (*n* = 5) were the most frequently used (Appendix S8). A significant decline in EXP was observed in 9 data points (50%), with reductions of up to 30% during lockdowns lasting 6 to 40 weeks. Increases were reported in 3 data points, while 6 showed no significant changes.

#### Speed

Seven studies evaluated speed [34, 36–41], yielding 17 data points (9 in males, 3 in females, 5 in mixed gender groups). The most frequently used tests were the 30-meters and 50-meters sprint tests, administered across 4 and 7 data points respectively, while repeated sprints (2 × 10 m and 4 × 10 m) were featured in 6 data points (Appendix S8). Decreases in speed performance were observed in 11 data points (65%), with reductions of up to 9.5% during lockdowns lasting 13 to 40 weeks. Five data points showed increases of up to 13%, and one data point indicated no significant change.

#### Motor Skills (agility, Balance, flexibility)

The influence of lockdown on agility [34, 37, 39] and flexibility [37, 38, 41] was investigated in 7 data points, and 3 studies each. Agility studies were most conducted on male participants (*n* = 5), while flexibility principally on mixed-gender groups (*n* = 3). Agility showed a predominant decline (*n* = 4; 57%), primarily reported by Alvurdu et al. [34]. Flexibility improved in 5 data points (72%), mostly in mixed-gender groups, while balance declined in 2 data points from Pombo et al. [39].

### Effect of Vacations on Physical Fitness Components

Sixteen studies were included [42–57], providing 68 data points. Participants were aged 6 to 17 years, with sample sizes ranging from 17 to 2,279. The gender distribution among data points was balanced: 19 in males, 19 in females, and 30 in mixed-gender groups. The duration of school vacations ranged from 3 to 24 weeks, and all studies focused on summer vacations (Fig. 3, Panel B).

#### Cardiorespiratory Fitness

All sixteen studies [42–57] yielded 31 data points assessing CRF. Most data points (*n* = 19) were obtained from mixed-gender groups, while 6 were from males and 6 from females. The most frequently assessed outcomes were VO₂max (*n* = 12), number of laps of different distances (*n* = 15), and distance covered (*n* = 4) (Appendix S3). The PACER test was the most used method (*n* = 15) (Appendix S8). A decrease in CRF was observed in 8 data points (26%), with reductions ranging from 4 to 16% during vacations lasting 10 to 20 weeks. Increases were reported in 3 data points (10%), but the majority (*n* = 20; 64%) showed no significant changes.

#### Muscle Strength

A single study conducted by Aphamis et al. [55] assessed muscle strength using the handgrip test, yielding 4 distinct data points for analysis (2 in males and 2 in females) (Appendix S8). No significant changes in MS were observed across these data points.

#### Power-strength

Four studies [44, 48, 50, 53] investigated the effect of summer vacations on power-strength, providing 8 data points (2 in males, 2 in females, and 3 in mixed-gender groups). Three assessment methods were used: sit-ups within a 30-second, 40-second, or 1-minute period (*n* = 6), 40-second push-up test (*n* = 1), and push-up to failure test (*n* = 1) (Appendix S8). Most data points (*n* = 6; 75%) indicated no significant changes in power-strength, while 2 data points (25%) reported increases.

#### Explosive Strength or Power

Three studies [44, 53, 55]), contributed 7 data points assessing explosive power, with 3 in males, 3 in females, and 1 in mixed-gender groups. The standing broad jump (*n* = 5) and squat jump (*n* = 2) were the primary tests used (Appendix S8). Most data points (*n* = 5; 71%) showed no significant changes in EXP during vacations. One data point (14%) reported a decrease of 18% after 20 weeks of vacation, while another (14%) indicated an increase following 16 weeks of summer.

#### Speed

Three studies [44, 53, 55] provided 7 data points on speed, with 3 in males, 3 in females, and 1 in mixed-gender groups. Assessment methods included the 20-m sprint (*n* = 1), 30-m sprint (*n* = 2), and 10 × 5 m shuttle run (*n* = 4) (Appendix S8). Decreases in speed were observed in 3 data points (43%) after 20 weeks of vacation, while 4 data points (57%) showed no significant changes. No increases in speed were reported.

#### Motor Skills (agility, Balance, flexibility)

The impact of vacations on flexibility was investigated across 6 studies [44, 48, 50, 53, 55], yielding 9 data points. The sit-and-reach test (*n* = 8) and stand-and-reach test (*n* = 1) were used (Appendix S8). No changes were found in flexibility. Agility and balance showed significant declines in one study after 16 weeks of vacation [44].

### Effect of Training Cessation on Physical Fitness

A total of 36 studies [24, 25, 58–91] provided 239 data points to assess the impact of training cessation on physical fitness in children. Participants were aged 7 to 18 years, with sample sizes ranging from 7 to 256. Most assessments were conducted on males (*n* = 150), followed by mixed-gender groups (*n* = 62) and females (*n* = 27). The duration of training cessation varied from 2 to 24 weeks (Fig. 3, Panel C).

#### Cardiorespiratory Fitness

Thirteen studies [59–61, 66, 69, 70, 74, 75, 78, 81, 85–87] provided 23 data points on CRF (14 in males, 3 in females and 6 in mixed gender). The most frequently used tests were the 20-m shuttle run (*n* = 10) and the Yo-Yo Intermittent Recovery Test (*n* = 5) (Appendix S8). CRF decreased in 12 data points (52%), with reductions of up to 21% for periods of 3 to 24 weeks. No significant changes were observed in 9 data points, while increases were reported in 2 data points by D’souza and Avadhany [75].

#### Muscle Strength

Twenty-one studies [24, 59, 62–64, 68, 69, 73, 75, 77, 79, 80, 82, 83, 85–91] contributed 69 data points on MS, with 41 in males, 5 in females, and 23 in mixed-gender groups. A wide range of tests was used (*n* = 32), with medicine ball throwing (*n* = 18) and leg press (*n* = 6) being the most common (Appendix S8). No significant changes were found in 40 data points (58%), while 25 data points (36%) showed declines of up to 30% over training cessation periods of 4 to 16 weeks. Increases were reported in 4 data points (6%) in D’souza and Avadhany [75].

#### Power-strength

Eight studies [24, 66, 67, 69, 71, 75, 78, 87] provided 24 data points on power-strength, with 15 in mixed-gender groups, 6 in males, and 3 in females. The tests assessed either strength maintenance (*n* = 22) or speed maintenance (*n* = 2). Decreases in power strength, ranging from 3 to 64%, were observed in 12 data points (50%) over periods of 3 to 12 weeks. The remaining 12 data points (50%) showed no significant changes, and no increases were reported.

#### Explosive Strength or Power

Twenty-one studies [24, 25, 58, 60, 62, 64, 69, 71–73, 77–80, 83–89, 91] provided 59 data points on explosive strength or power, with 41 in males, 9 in females, and 9 in mixed-gender groups. EXP was assessed through 12 different tests, with standing broad jump and countermovement jump being the most common (Appendix S8). Most data points (*n* = 36; 61%) showed no significant changes, while declines were reported in 21 data points (36%), with reductions of up to 30% for periods of 4 to 16 weeks. An increase was observed in one data point (3%) [84].

#### Speed

Twelve studies [25, 60, 64, 69, 74, 78, 80, 83–86, 91] provided 34 data points on speed, predominantly in males (*n* = 32). Decreases in speed were observed in 18 data points (53%), with reductions of up to 11% during training cessation periods of 4 to 16 weeks. No significant changes were observed in 14 data points (41%), while increases were reported in 2 data points.

#### Motor Skills (agility, Balance, flexibility)

The effect of training cessation was most studied on balance (*n* = 11; 4 studies [60, 69, 71, 79]) and flexibility (*n* = 11; 5 studies [58, 65, 67, 69, 76]), then on agility (*n* = 8; 2 studies [60, 69]). Males were the most assessed gender group (*n* = 2; 50%). Data points indicated declines in agility (*n* = 6; 75%), balance (*n* = 4; 36%), and flexibility (*n* = 2; 18%). No increases were reported in any of the motor skills assessed.

### Effect of Moderators

Cardiorespiratory fitness, muscle strength, explosive power and speed were the selected key parameters where sufficient data were available (> 50 data points).

#### Effect of Age

**Fig. 4:**
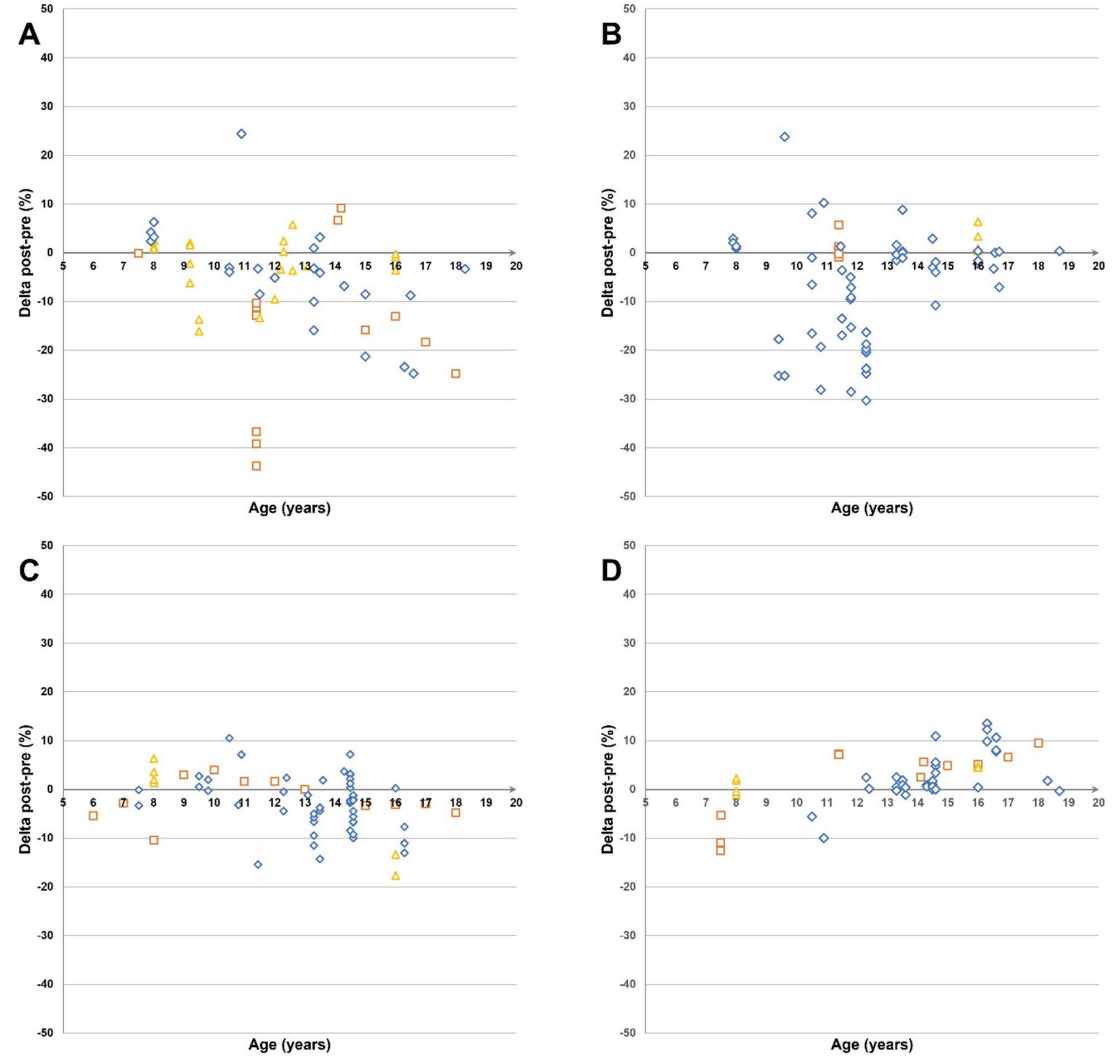
Pre- to post-disruption changes in physical fitness components across participants age **(Panel A)** In cardiorespiratory fitness. **(Panel B)** In muscle strength. **(Panel C)** In explosive power. **(Panel D)** In speed

Regardless of the nature of the disruption, our analysis suggests that age influences CRF, MS, and speed differently. For CRF, the magnitude of decreases tends to escalate with age, indicating that older children and adolescents are more affected by disruptions. In contrast, younger children (before 12–13 years old) experience more pronounced declines in MS. Speed decreases are only observed in participants aged 13–14 years and older, suggesting different age sensitivities for these fitness components (Fig. 4).

#### Effect of Gender

The analysis did not reveal any consistent trends regarding the impact of disruptions based on gender. No clear patterns were identified across the different types of disruptions. A graphical analysis of the effect of gender is provided in Appendix S9.

#### Effect of Duration of the Disruption

**Fig. 5:**
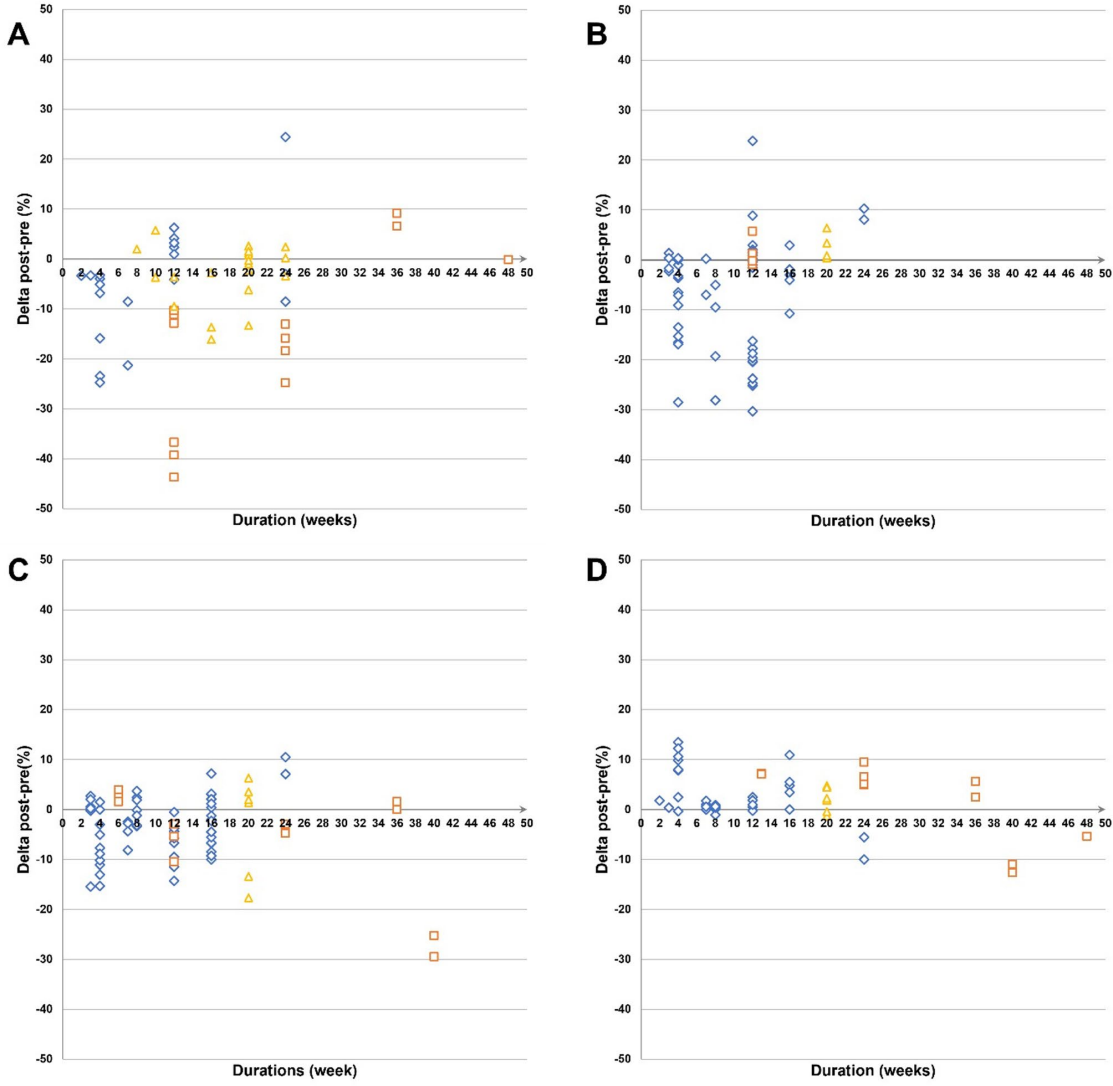
Pre- to post-disruption changes in physical fitness components across duration of the disruptions **(Panel A)** In cardiorespiratory fitness. **(Panel B)** In muscle strength. **(Panel C)** In explosive power. **(Panel D)** In speed

The duration of the disruption did not seem to influence physical fitness outcomes. Decreases in physical fitness were similar regardless of whether the disruption was short (starting at 2 weeks) or extended (Fig. 5).

#### Effect of Baseline VO_2_max

**Fig. 6:**
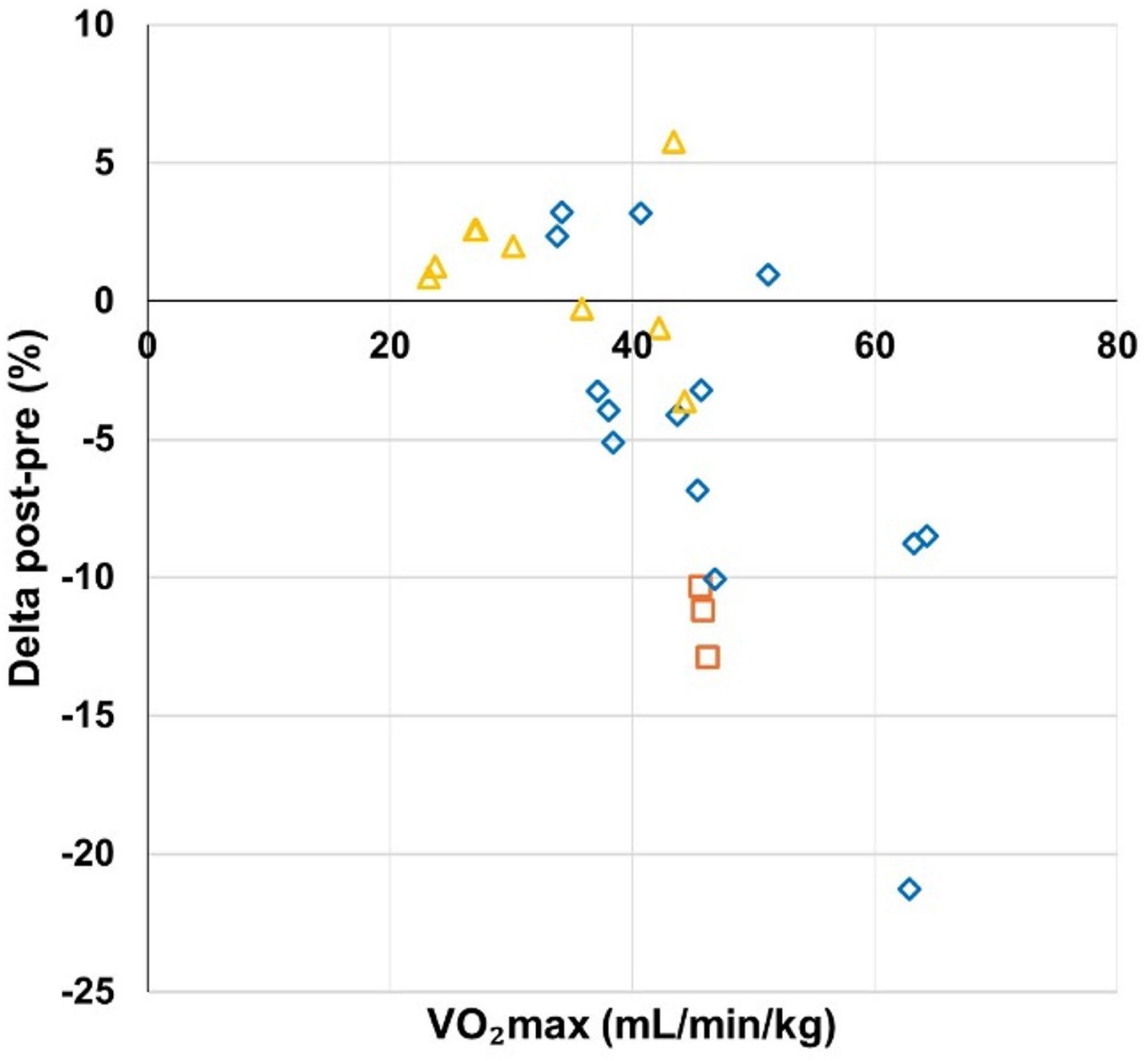
VO_2max_ baseline levels and pre- to post-disruption changes

Due to the high variability in tests and parameters across studies, the analysis was restricted to CRF measured by VO2max or VO2peak assessment tests (23 data points) for comparability. The results indicated that higher initial VO2max levels are associated with more substantial declines in CRF following disruptions. These findings are illustrated in Fig. 6.

## Discussion

This systematic scoping review aimed to (1) map the effects of disruptive situations—lockdown, vacations, and training cessation—on the physical fitness components of children and adolescents, and (2) identify potentially moderators such as the duration of the disruption, age, gender, and baseline levels of the participants. A total of 60 longitudinal studies that explored the impact of disruptive situations on the physical fitness of young individuals were systematically identified. Most of the included studies focused on the effects of training cessation (*n* = 36) [24, 25, 58–91], with a limited exploration of the impact of vacations (*n* = 16) [42–57] and of lockdown (*n* = 8) [34–41] on a young population.

The impact of various disruptions on cardiorespiratory fitness reveals distinct patterns (Fig. 3, Panel A). During lockdowns, a notable decline in CRF was observed. This is consistent with the study by Silva et al. (2022) [8] which noted that changes in aerobic capacity over time are influenced by physical activity and with findings from Paterson et al. (2021) [6] which indicate that lockdowns increased sedentary behavior and decreased physical activity levels. Therefore, restrictions due to lockdown on outdoor activities and organized sports reduced opportunities for maintaining physical activity levels, further influencing changes in aerobic capacity over time [8, 94]. This review found no data on lockdown effects on CRF in children, complicating age-related conclusions. However, it seems lockdown could induce decreases in CRF in both pre-adolescents and adolescents. Graphical analysis also did not reveal clear gender differences in response to lockdown, nor did it identify a correlation between lockdown duration and the magnitude of CRF decline.

In contrast, the impact of vacations on CRF varied among studies: 64% reported no significant changes, 26% observed decreases, and 10% noted increases. During vacations, children’s activities become unstructured without the school environment, leading to varied experiences and outcomes for everyone [18]. While some studies, like Fu et al. (2020) [46] or Watson et al. (2023) [95], reports declines in physical activity during vacations, others, such as Volmut et al. (2021) [96], find increases in outdoor activities and higher physical activity levels. These inconsistencies highlight a critical limitation in the literature: the inconsistent measurement of physical activity and sedentary during vacations. Furthermore, no significant effects of age, gender, or vacation duration on CRF were found, but the limited scope of studies limits broader generalizations.

CRF decreased in 52% of studies during training cessation, with 39% showing no significant changes, reflecting variability. Unlike lockdowns, training cessation may allow for other physical activities, explaining some of this inconsistency [7]. Furthermore, it primarily affects active youth, with higher baseline fitness levels, which is linked to greater CRF declines in adults [97]. For example, in young soccer players, those with higher VO_2_max values experienced declines exceeding 20% [25] (Fig. 6). Age also plays a role, as older adolescents tend to experience more significant CRF declines (Fig. 4). While VO_2_max increases during childhood, it tends to plateau after puberty [98], making adolescents more vulnerable to CRF loss when training stops. In contrast, younger children may see less decline due to maintaining spontaneous activity whereas sedentary behavior and inactivity peak during adolescence [99]. No significant gender differences were observed, and the length of detraining did not correlate with greater declines, consistent with findings that CRF drops as soon as training stops [71].

The effects on CRF are influenced by the specific context of disruptions. Lockdowns, with their broad restrictions on daily activities, led to significant CRF declines. Vacations showed varied effects, likely due to individual differences in unstructured activities and inconsistent measurement of movement behaviors. Training cessation mainly affected active individuals, with more pronounced declines linked to higher baseline fitness levels and older age.

The evolution of muscle strength and power-strength during disruptions remains relatively stable, with 57–100% and 50–78% of data points reporting no significant changes, respectively. MS and PS improve during childhood and adolescence because of natural growth (neuromuscular system) and maturation (muscular system) processes [100]. While environmental factors and physical activity influence these developments, they are largely driven by ongoing neuro-muscular growth, which mitigates the negative effects of disruptions [101]. Consequently, the stability of MS and PS is attenuated by the continuous growth and maturation, which attenuates the adverse effects of disruptive situations.

The most significant impacts on MS occur between ages 10 and 13, just before puberty (Fig. 4). In childhood, physical development is primarily neural, whereas adolescence involves both neural and hormonal changes [101]. Hormonal adaptations, which can occur even with low physical activity, lead to rapid gains in muscle mass and strength post-puberty [102], helping offset the effects of reduced activity or training cessation [101, 103]. No significant gender differences were observed, likely due to the limited data on girls (only 9 data points), making it difficult to draw conclusive gender comparisons. Duration also did not seem to influence the effects, as the magnitude of changes was consistent regardless of disruption length. Additionally, the wide variety of test methods (32 different tests) used to measure muscle strength complicates comparisons (Appendix S8). Future research should focus on assessing the impact of disruptions on muscle strength and power-strength in girls, using standardized testing methods.

The impacts of various disruptions on speed and explosive power reveal questioning patterns. Both qualities rely heavily on the neuromuscular system [104]. However, their responses to disruptions vary in magnitude depending on the nature of the disruptive situation. Explosive strength or power power seems more resilient than speed, particularly during lockdowns, where 65% of data points reported decreases in speed and 50% in explosivity. The severe impact of lockdown is likely due to the sudden cessation of school and structured physical activity, along with limited opportunities for high-intensity activities [6]. However, responses were more balanced over the other two disruptive situations, as declines in speed were less pronounced in vacations and training cessation (43% and 53%, respectively) and a major portion of data points reported no significant changes in explosive strength or power (72% and 61%, respectively). This may be due to the less restrictive nature of these disruptions, which allowed for the maintenance of unstructured physical activity and active situations [96].

Speed primarily relies on the neuromuscular system, which requires consistent, high-intensity training to maintain the coordination and efficiency of nerve impulses and muscle contractions [105]. Without regular practice, these neuromuscular connections weaken, leading to a more pronounced decline in speed [106]. In contrast, explosivity depends more on the muscular system’s strength and power. While neuromuscular coordination is also important, the primary factor is the muscle’s ability to generate force quickly. Muscle power can be more resilient to periods of detraining, as it does not degrade as rapidly as neuromuscular efficiency [89].

Age seems to moderate speed’s response to disruptions, with most declines occurring after 12–13 years, likely due to interactions between growth, maturation, and training [107]. Adolescents, especially those post-puberty and with higher training levels, seem more susceptible to declines in fitness when training is disrupted. However, the data primarily focus on adolescents, limiting conclusions. No age effects were observed for explosive strength or power, and no gender or duration effects were noted across the disruptions.

The following discussion aims to explain the observed effects of disruptions on flexibility, agility, and balance though conclusions are cautious due to the limited data available. Disruptions seem to have minimal impact on flexibility, with 89% of data points during vacations and 82% during training cessation showing maintenance. This stability is likely due to the strong genetic influence on flexibility, as noted by Massidda et al. [108] Interestingly, 72% of studies during COVID-19 lockdowns reported improvements in flexibility, particularly in China, where authorities promoted indoor physical exercises to maintain fitness, as noted by Li and Cheong [37]. As described a decade ago [101], agility remains under-researched in youth, with only 15 data points available. Most of these indicate decreases in agility across all disruptions: 67% during lockdown, 100% during vacations, and 75% during training cessation. Evidence from training studies suggests that agility is trainable in youth, and is therefore sensitive to the cessation of occasions to train this component, which likely explains these decreases [109]. Balance, less influenced by genetics and more by person-specific environmental factors [105], showed decreases in many data points. This trainable aspect of the component could explain why many data points reported decreases in balance.

### Strengths and Limitations

Key strengths of this scoping review include a systematic search strategy and quality assessment. The review uniquely groups three disruptive scenarios—lockdowns, vacations, and training cessation—to examine their effects on physical fitness, marking to our knowledge the first such analysis.

However, the wide variability in testing methods across studies complicates comparisons, thereby limiting the strength of the conclusions. The small number of studies, especially those involving girls, further challenges the ability to draw definitive conclusions. Additionally, the lack of control for key health behaviors like physical activity, sedentary behaviors, and sleep during disruptions weakens the level of evidence by limiting our ability to fully assess the impact of these situations.

## Conclusion

Disruptive situations like lockdowns, vacations, and training cessations have impacted the physical fitness of children and adolescents, with effects varying by disruption type. Cardiorespiratory fitness generally declined, particularly among older adolescents, highlighting the need for regular physical activity to maintain heart and lung health. In contrast, muscular fitness—encompassing strength, power, speed, and explosive strength or power—remained relatively stable, likely due to ongoing growth and maturation. Given the heightened sensitivity of older adolescents to these disruptions, physical activity guidelines should focus on this group.

The review reveals a need for more longitudinal studies to better understand the long-term impacts of disruptions on physical fitness. It also underscores the importance of addressing the gender gap by prioritizing research on girls. Assessing baseline fitness levels and controlling movement behaviors during disruptions are crucial for enhancing the accuracy and applicability of findings on how these situations affect physical fitness in youth.

## Electronic Supplementary Material

Below is the link to the electronic supplementary material.

Supplementary Material 1: Appendix S1 - Search strategy for each database.

Supplementary Material 2: Appendix S2 - Data extraction of studies on lockdown

Supplementary Material 3: Appendix S3 - Data extraction of studies on school vacations

Supplementary Material 4: Appendix S4 - Data extraction of studies on training cessation

Supplementary Material 5: Appendix S5 - Quality assessment of pre-post studies with no control groups

Supplementary Material 6: Appendix S6 - Quality assessment of controlled intervention studies

Supplementary Material 7: Appendix S7 - Quality assessment of cross-sectional and cohort studies

Supplementary Material 8: Appendix S8 - Summary of tests employed in studies and their frequency of use

Supplementary Material 9: Appendix S9 - Effect of disruptions in boys, girls and mixed groups

Supplementary Material 10

## Data Availability

The data used to support the findings of this study are included within the article and the supplementary information file.

## Abbreviations

CRF: Cardio respiratory fitness
EXP: Explosive strength or power
MS: Muscle strength
PS: Power-strength
