# Supplementary figures and images for: Exploring the Effects of Lifestyle Disruptions on Physical Fitness in Children and Adolescents: a Systematic Scoping Review
Source: Sports Med Open. 2025 Jun 7;11:65. doi: 10.1186/s40798-025-00883-0 (PMC12145346; doi:10.1186/s40798-025-00883-0)

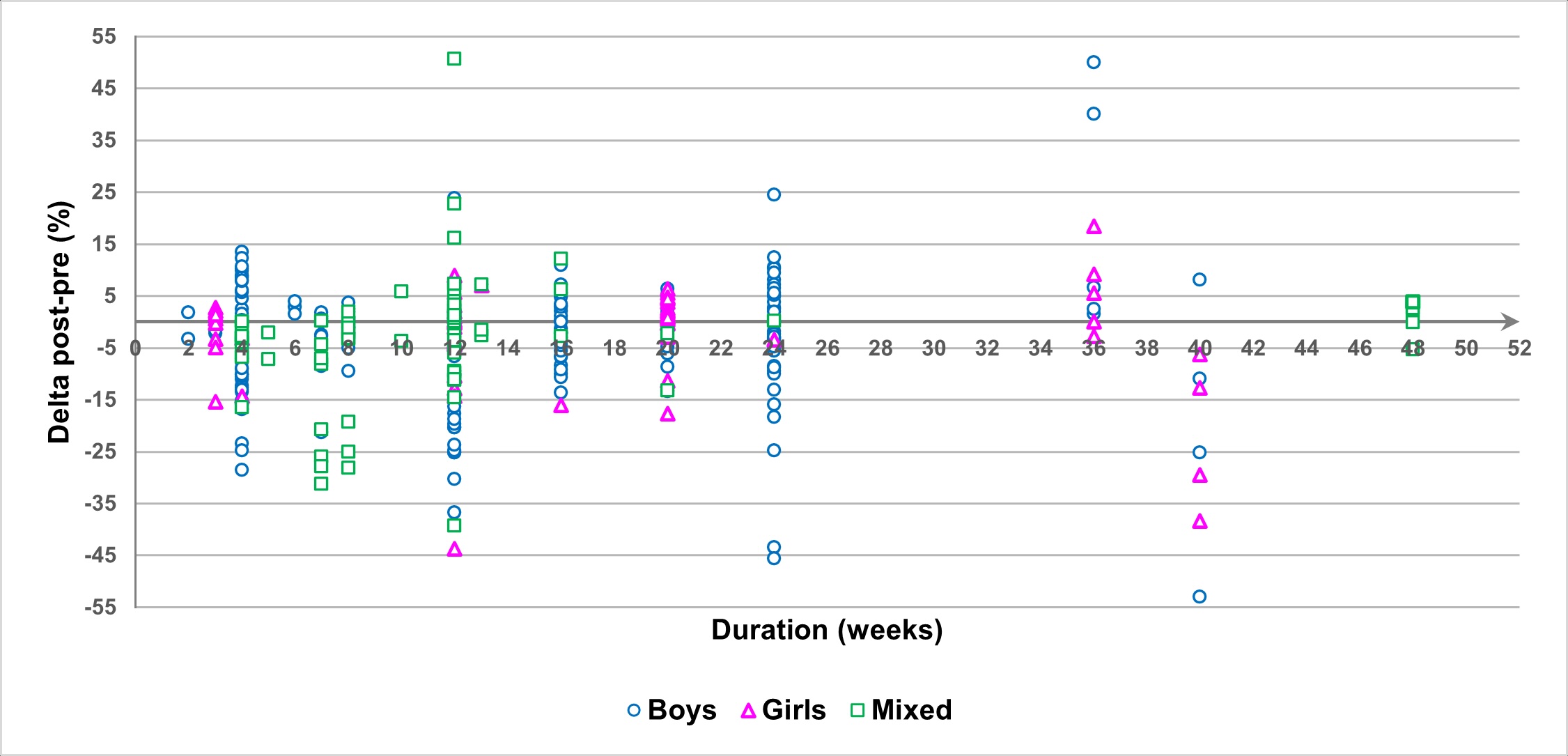

Supplement: Supplementary file 10 — Supplementary Material 10 [file 40798_2025_883_MOESM10_ESM.jpg]
