# Supplementary material for: Exploring the Effects of Lifestyle Disruptions on Physical Fitness in Children and Adolescents: a Systematic Scoping Review
Source: Sports Med Open. 2025 Jun 7;11:65. doi: 10.1186/s40798-025-00883-0 (PMC12145346; doi:10.1186/s40798-025-00883-0)
Supplement: Supplementary file 1 — Supplementary Material 1: Appendix S1 - Search strategy for each database. [file 40798_2025_883_MOESM1_ESM.docx]

**Cover letter**

***Subject :* Submission of a systematic scoping review**

**La Garde, November 13^th^**

**Dear Editor-in-Chief,**

I am pleased to submit our manuscript titled **“Exploring the Effects of Lifestyle Disruptions on Physical Fitness in Children and Adolescents: A Systematic Scoping Review”** for consideration in **Sports Medicine-Open**.

Maintaining good physical fitness is crucial for a healthy lifestyle. In younger populations, however, fitness routines can be disrupted by factors such as school closures, interruptions in training, or unexpected events like the COVID-19 lockdown.

Our study presents a comprehensive systematic scoping review investigating the impact of various lifestyle disruptions, including lockdowns, school vacations, and periods of training cessation, on the physical fitness of children and adolescents. We examine the effects on several components of physical fitness, including cardiorespiratory fitness, muscle strength, explosive power, and more. The review emphasizes the differential impacts based on age, sex, baseline fitness levels, and the duration of the disruptions.

This research is particularly relevant given the global emphasis on youth physical fitness and the lasting consequences of the COVID-19 pandemic. Maintaining physical fitness is a major challenge for the public health. Our conclusion showed that cardiorespiratory fitness generally declined, particularly among older adolescents, highlighting the need for regular physical activity to maintain heart and lung health. In contrast, muscular fitness—encompassing strength, power, speed, and explosivity—remained relatively stable, likely due to ongoing growth and maturation. Given the heightened sensitivity of older adolescents to these disruptions, physical activity guidelines should focus on this group, particularly in girls.

We believe these insights will be of interest to the readers of **Sports Medicine**, who are at the forefront of exercise science, pediatric sports medicine, and health policy development.

We confirm that this manuscript is original, has not been previously published, and is not under consideration elsewhere. All authors have read and approved the manuscript, and we have no conflicts of interest to disclose.

We believe this paper aligns well with the scope of **Sports Medicine-Open**, offering a valuable synthesis of evidence that can inform both research and practice in pediatric exercise science. Our findings may help shape future strategies to mitigate the adverse effects of unexpected lifestyle changes on youth fitness.

We appreciate your time and consideration of our manuscript for publication. Please do not hesitate to contact me if you require any additional information.

Best regards,

Pr Pascale Duché
